# Tacrolimus‐Related Posterior Reversible Encephalopathy Syndrome in a Postlung Transplant Patient: A Case Report

**DOI:** 10.1155/crcc/2365836

**Published:** 2025-09-10

**Authors:** Lucia C. Silva, Eduardo Tuta-Quintero, Fabio Varón-Vega

**Affiliations:** ^1^ Department of Critical Care, Fundación Neumológica Colombiana, Fundación Cardio Infantil-Instituto de Cardiología, Bogotá, Colombia, nosm.ca; ^2^ School of Medicine, Universidad de La Sabana, Chía, Colombia, unisabana.edu.co; ^3^ Department of Critical Care and Lung Transplant, Fundación Neumológica Colombiana, Fundación Cardio Infantil-Instituto de Cardiología, Bogotá, Colombia

**Keywords:** lung transplantation, posterior leukoencephalopathy syndrome, tacrolimus

## Abstract

We present the case of a 58‐year‐old woman with fibrotic hypersensitivity pneumonitis, without significant comorbidities, who developed posterior reversible encephalopathy syndrome (PRES) 7 days after a bilateral lung transplant. Initial symptoms included hypertension, headache, nausea, vomiting, and cortical blindness. Although no seizures were observed, the electroencephalogram revealed occipital epileptic discharges. Brain magnetic resonance imaging confirmed the diagnosis of PRES, showing a typical pattern of vasogenic edema in the corticosubcortical occipital regions. Tacrolimus was discontinued, and antihypertensives and anticonvulsants were initiated, resulting in complete neurological recovery within 4 days. After 16 days without calcineurin inhibitor, cyclosporine was introduced with no recurrence of the neurological condition.

## 1. Background

Posterior reversible encephalopathy syndrome (PRES) is an acute brain condition resulting from failures in cerebral vascular autoregulation and/or endothelial dysfunction [1, 2]. These alterations lead to disruption of the blood–brain barrier and vasogenic cerebral edema. In some cases, PRES may be complicated by infarcts or hemorrhages [2, 3]. This syndrome can present in various contexts, including patients undergoing solid organ transplantation (SOT) or allogeneic bone marrow transplantation, and is often associated with the use of immunosuppressive medications, particularly calcineurin inhibitors (CNIs) [4–6].

Although CNI‐related PRES was initially documented in populations in the United States and Europe [1, 3, 4], this condition can occur in diverse populations and clinical contexts. Documenting this case not only broadens the understanding of PRES in the local population but also highlights the importance of being alert to this potential complication in patients receiving immunosuppressive therapies, thereby contributing to the improvement of monitoring and management protocols in lung transplants and other similar clinical scenarios [2, 3, 6]. We report a clinical case of a patient with fibrotic hypersensitivity pneumonitis who underwent a lung transplant and developed PRES associated with tacrolimus immunosuppression.

## 2. Case Report

A 58‐year‐old woman with fibrotic hypersensitivity pneumonitis, who had experienced symptomatic and functional deterioration despite treatment with azathioprine (50 mg every 12 h), prednisone (5 mg daily), and calcium with vitamin D supplements, was hospitalized for a lung transplant. She had Group 3 pulmonary hypertension without right ventricular dysfunction as a comorbidity. Her blood type was O+, with a history of multiparity (five pregnancies) and a negative HLA Class I and II antibody panel.

She underwent a sequential double lung transplant from an ABO‐identical brain‐dead donor, without the need for extracorporeal circulation. The patient was extubated after 48 h and received intermittent noninvasive ventilation for an additional 72 h, without surgical complications. Postoperative prophylaxis included antibiotics (betalactam and betalactamase inhibitor for 5 days), antiparasitic (albendazole for 3 days), antifungals (oral nystatin and vaginal clotrimazole for 5 days), and antiviral (ganciclovir starting on the second postoperative day).

Initial immunosuppression consisted of methylprednisolone (1 g IV presurgery and on the first postoperative day), followed by 125 mg every 8 h IV on the second day, and prednisone (1 mg/kg daily starting on the third day). Additionally, mycophenolate mofetil (1 g presurgery, followed by 750 mg every 12 h starting on Day 2) and tacrolimus (3 mg every 12 h from the third day) were administered, with levels on Days 5 and 7 being 7.7 and 7.5 ng/mL, respectively.

**Figure 1:**
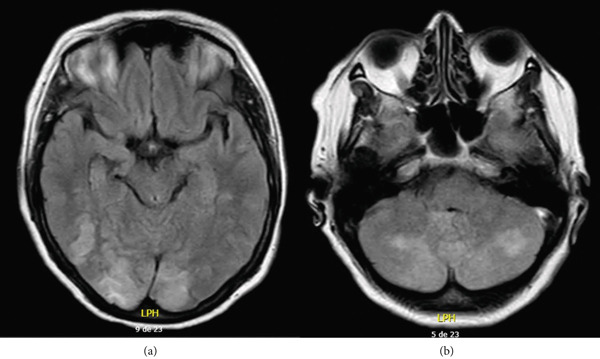
Hyperintense lesions in the occipital and cerebellar lobes. Note: axial slices from a brain magnetic resonance imaging in fluid‐attenuated inversion recovery sequence showing hyperintense corticosubcortical lesions, bilaterally located in the (a) occipital and (b) posterior cerebellar lobes.

On Postoperative Day 7, the patient developed blurry vision that progressed within 2 h to bilateral amaurosis, accompanied by headache, nausea, and vomiting. Physical examination showed elevated blood pressure (170/100 mmHg) and bilateral amaurosis without other neurological deficits. Brain MRI revealed two lesions in the occipital lobes, located in the posterior and medial regions, with corticosubcortical involvement, high intensity on T2 and FLAIR (see Figure 1a), without representation on T1, and without diffusion restriction. Additionally, two oval lesions were observed in the posterior cerebellar lobes with similar characteristics (see Figure 1b). Visual evoked potentials were normal, and video telemetry showed frequent focal epileptiform activity in the posterior quadrants with a right‐sided predominance.

**Figure 2:**
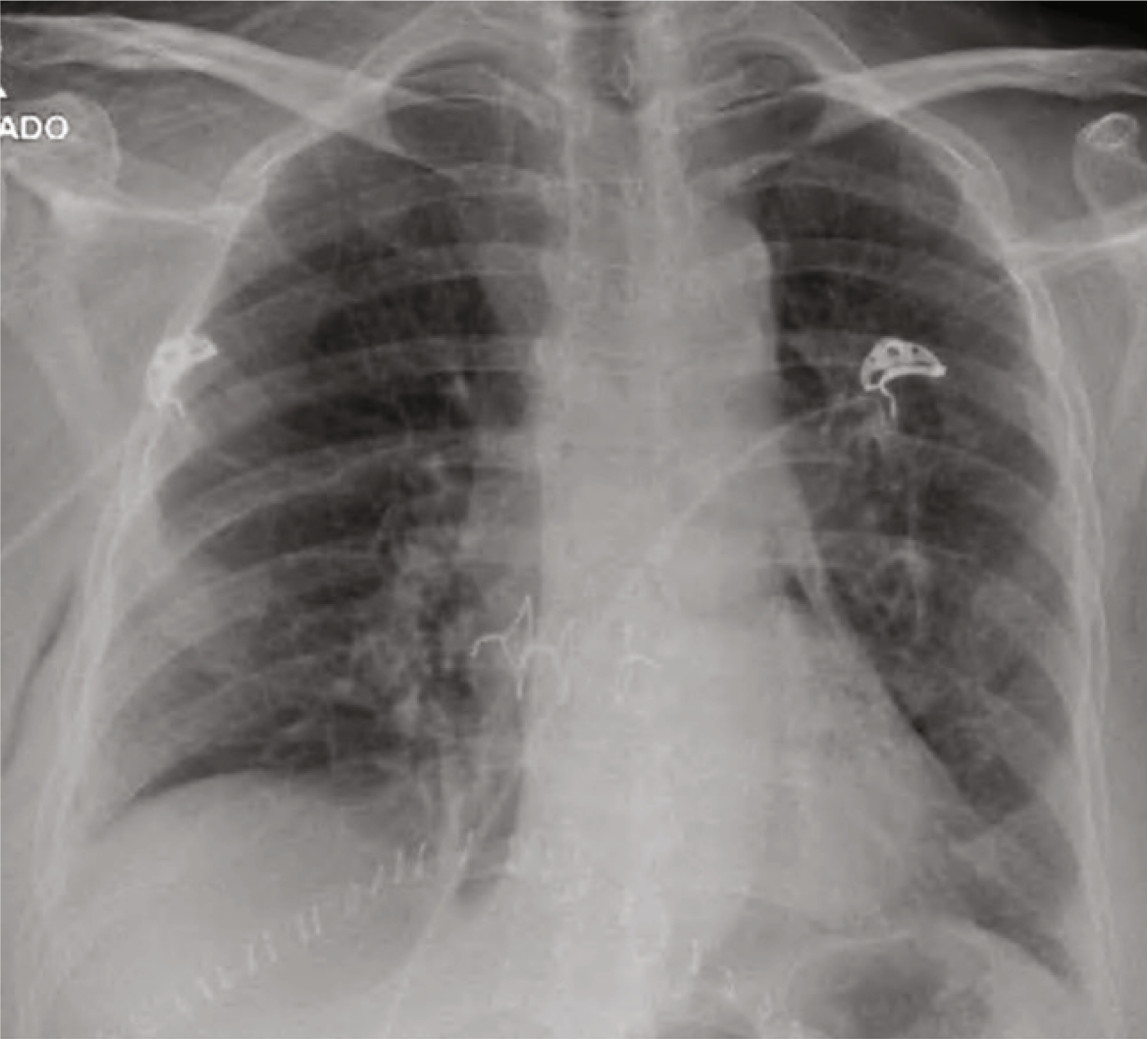
Chest x‐ray on postoperative.

PRES was diagnosed, attributed to tacrolimus. Treatment included antiemetics, antihypertensives, and lacosamide, with tacrolimus being immediately discontinued. By Day 8, the patient exhibited mild confusion, which resolved by Day 9, with progressive improvement in visual symptoms. On Day 11, the patient achieved full neurological recovery, and the lung transplant showed favorable progress, as confirmed by chest x‐ray findings (see Figure 2). Due to the need to discontinue tacrolimus and to prevent acute rejection, thymoglobulin (1 mg/kg) was administered on Postoperative Days 8, 14, and 21. From Day 23, cyclosporine (2.5 mg/kg every 12 h) was started, with adjustments based on C2 levels. The patient remained asymptomatic both respiratory and neurologically on Postoperative Day 36.

## 3. Discussion

In our case, PRES presented early, on the seventh postoperative day, while tacrolimus levels were still subtherapeutic (7.5 ng/mL compared to a target of > 10 ng/mL). The immediate discontinuation of the CNI resulted in a rapid neurological improvement. Subsequently, cyclosporine was started, following common practice reported in the literature, without observing a recurrence of PRES. During a 16‐day interval, no CNI was administered, and repeated doses of thymoglobulin were used to prevent acute rejection. It is plausible that this CNI‐free interval contributed to cerebral tissue repair and recovery, although it is not possible to determine with certainty the specific impact of this strategy on PRES prevention.

Despite tacrolimus levels being within the lower therapeutic range, the temporal association between drug initiation and symptom onset, the prompt resolution after drug withdrawal, and the absence of recurrence upon switching to cyclosporine support a causal link. This reinforces prior reports indicating that PRES may occur even at subtherapeutic or therapeutic CNI levels [7, 8].

The clinical presentation of PRES in our patient largely matched expectations; however, there are three noteworthy particularities. First, the presence of elevated blood pressure, while common at the onset of the clinical picture, is not universal, and up to 31% of patients may be normotensive. Second, although visual disturbances are frequent, cortical blindness, as experienced by our patient, is reported in only 5.6% of cases. Third, while seizures can occur in up to 77% of patients [7, 8], our patient did not show clear ictal semiology. However, the electroencephalogram revealed focal epileptiform discharges in the areas affected, as seen on imaging. These subclinical epileptic discharges are rarely documented in case series but may precede clinical seizures [2, 7, 8]. Early detection of these discharges via telemetry and early initiation of anticonvulsant treatment may have contributed to preventing the clinical manifestation of seizures in our patient. It highlights the utility of electroencephalography monitoring, which can detect cortical hyperexcitability, identify subclinical seizures, and point to other causes of encephalopathy, particularly in patients presenting with visual symptoms or altered mental status, and could aid in identifying high‐risk individuals and tailoring anticonvulsant management [9].

PRES associated with SOT and CNI use is a rare but recognized complication. In large cohorts such as Bartynski’s, it was observed in 0.49% of patients (21 cases in 4222 transplants over 8 years) [7], with a similar incidence across different types of SOTs [7] and has been documented with both tacrolimus and cyclosporine [8]. A Cochrane review, which included three randomized clinical trials comparing both CNIs in lung transplantation, revealed that only one of these studies reported neurotoxicity as an adverse effect. In that study, three of 124 patients receiving tacrolimus developed neurotoxicity compared to none of the 125 patients receiving cyclosporine. This difference was not statistically significant (*p* = 0.12) [10]. Furthermore, tacrolimus serum levels do not seem to correlate with the occurrence of PRES. As in Bartynski’s cohort, 82% of PRES cases had tacrolimus levels within the therapeutic range [7].

Although CNI‐related PRES was initially described in the US and European populations [1], it is an idiosyncratic reaction that can manifest in other populations. This is the first case of PRES recorded at our center in Bogotá, which has performed 70 lung transplants since its inception. According to a preliminary literature review, three cases of PRES have previously been documented in Colombia in SOT patients: two in heart transplants [2] and one in a kidney transplant [4], all associated with tacrolimus.

A review that aggregated data from various reports and case series of PRES associated with SOT and CNIs identified a total of 71 cases [8]. This review showed that PRES typically begins early, with a median of 17 days posttransplant, although 7.3% of cases may present more than a year after transplantation. While the literature emphasizes discontinuing the causative agent as a fundamental pillar of treatment, the use of CNIs as a cornerstone of posttransplant immunosuppression presents a clinical challenge. In the analysis of this case series, 26.8% of patients switched to another CNI; 22.5% reduced the dose of the same CNI; 16.9% replaced the CNI with an mTOR inhibitor, mycophenolate, or steroid; 19.7% temporarily discontinued the CNI; 9.9% permanently discontinued the CNI without substitution; and 4.2% made no changes. Of these cases, 89.3% experienced complete recovery, while 10.7% recovered without sequelae. The resolution of clinical signs had a median of 6 days, and imaging resolution had a median of 30 days [7, 8].

Although the patient recovered completely without neurological sequelae, no follow‐up brain MRI was performed to document radiological resolution. This is a limitation, as imaging could confirm reversibility of the lesions and rule out structural complications. Nevertheless, the favorable clinical course and absence of recurrence support the diagnosis and successful management strategy.

In conclusion, this case highlights the successful management of a 58‐year‐old woman with fibrotic hypersensitivity pneumonitis who underwent a sequential double lung transplant. Despite the development of PRES attributed to tacrolimus, prompt diagnosis and treatment, including discontinuation of tacrolimus and the use of alternative immunosuppression, led to complete neurological recovery and favorable graft outcomes. This case underscores the importance of vigilance for PRES as a potential complication in transplant recipients and the need for tailored immunosuppressive strategies to optimize patient and graft outcomes.

## Conflicts of Interest

The authors declare no conflicts of interest.

## Funding

This study was funded by the Fundación Neumológica Colombiana and the Fundación Cardioinfantil ‐ Instituto de Cardiología (10.13039/501100008727).

## Data Availability

The data that support the findings of this study are available from the corresponding author upon reasonable request.

## References

[1] Hinchey J. , Chaves C. , Appignani B. , Breen J. , Pao L. , Wang A. , Pessin M. S. , Lamy C. , Mas J. L. , and Caplan L. R. , A Reversible Posterior Leukoencephalopathy Syndrome, The New England Journal of Medicine. (1996) 334, no. 8, 494–500, 10.1056/NEJM199602223340803, 2-s2.0-13344284635. 8559202

[2] Rodríguez-González M. J. , Calvo-Betancourt L. S. , and Echeverría-Correa L. E. , Tacrolimus-Associated Posterior Reversible Encephalopathy Syndrome, Revista Colombiana de Cardiología. (2016) 23, no. 1, 69.e1–69.e5.

[3] Silva F. A. , Díaz G. A. , Ruíz N. P. , Echeverría L. E. , and Ocampo M. D. , Síndrome de Encefalopatía Posterior Reversible en un Paciente Trasplantado: Reporte de Caso, Acta Neurológica Colombiana. (2011) 27, no. 1, 45–54.

[4] Cadavid-Aljure D. , Caicedo-Paredes A. , Meza J. C. , Granados-Sánchez A. M. , Posada-Chávez J. G. , Mesa-Ramírez L. , and Schweineberg-López J. , Tacrolimus Associated to Posterior Reversible Atypical Encephalopathy Syndrome and Brain Haemorrhage in Renal Transplant Recipient, Nefrología. (2012) 32, no. 6, 861–863, 10.3265/Nefrologia.pre2012.Jul.11653, 2-s2.0-84873737162, 23169384. 23169384

[5] Jeelani H. , Sharma A. , and Halawa A. M. , Posterior Reversible Encephalopathy Syndrome in Organ Transplantation, Experimental and Clinical Transplantation. (2022) 20, no. 7, 642–648, 10.6002/ect.2021.0268. 35924741

[6] Geocadin R. G. , Posterior Reversible Encephalopathy Syndrome, The New England Journal of Medicine. (2023) 388, no. 23, 2171–2178, 10.1056/NEJMra2114482. 37285527

[7] Bartynski W. S. , Tan H. P. , Boardman J. F. , Shapiro R. , and Marsh J. W. , Posterior Reversible Encephalopathy Syndrome After Solid Organ Transplantation, AJNR. American Journal of Neuroradiology. (2008) 29, no. 5, 924–930, 10.3174/ajnr.A0960, 2-s2.0-43649099775. 18272559 PMC8128592

[8] Song T. , Rao Z. , Tan Q. , Qiu Y. , Liu J. , Huang Z. , Wang X. , and Lin T. , Calcineurin Inhibitors Associated Posterior Reversible Encephalopathy Syndrome in Solid Organ Transplantation, Medicine. (2016) 95, no. 14, 10.1097/MD.0000000000003173, 2-s2.0-84964614322, 27057842. PMC4998758 27057842

[9] Hobson E. V. , Craven I. , and Blank S. C. , Posterior Reversible Encephalopathy Syndrome: A Truly Treatable Neurologic Illness, Peritoneal Dialysis International. (2012) 32, no. 6, 590–594, 10.3747/pdi.2012.00152, 2-s2.0-84870994780, 23212858. 23212858 PMC3524908

[10] Treede H. , Glanville A. R. , Klepetko W. , Aboyoun C. , Vettorazzi E. , Lama R. , Bravo C. , Knoop C. , Aubert J. D. , and Reichenspurner H. , Tacrolimus and Cyclosporine Have Differential Effects on the Risk of Development of Bronchiolitis Obliterans Syndrome: Results of a Prospective, Randomized International Trial in Lung Transplantation, The Journal of Heart and Lung Transplantation. (2012) 31, no. 8, 797–804, 10.1016/j.healun.2012.03.008, 2-s2.0-84863337211. 22554673

